# Development of Conjugated Kefiran-Chondroitin Sulphate Cryogels with Enhanced Properties for Biomedical Applications

**DOI:** 10.3390/pharmaceutics15061662

**Published:** 2023-06-05

**Authors:** Hajer Radhouani, Cristiana Gonçalves, F. Raquel Maia, Eduarda P. Oliveira, Rui L. Reis, Joaquim M. Oliveira

**Affiliations:** 1 3B’s Research Group, I3Bs—Research Institute on Biomaterials, Biodegradables and Biomimetics, University of Minho, Headquarters of the European Institute of Excellence on Tissue Engineering and Regenerative Medicine, AvePark, Parque de Ciência e Tecnologia, Zona Industrial da Gandra, Barco, 4805-017 Guimarães, Portugal; cristiana.goncalves@i3bs.uminho.pt (C.G.); raquel.maia@i3bs.uminho.pt (F.R.M.); eduarda.oliveira@i3bs.uminho.pt (E.P.O.); rgreis@i3bs.uminho.pt (R.L.R.); miguel.oliveira@i3bs.uminho.pt (J.M.O.); 2 ICVS/3B’s—PT Government Associate Laboratory, 4805-017 Guimarães, Portugal

**Keywords:** biomedical device, carbodiimide-mediated coupling, characterization, chondroitin sulfate, cryogelation technique, kefiran, scaffold

## Abstract

Hydrogels based on natural polysaccharides can have unique properties and be tailored for several applications, which may be mainly limited by the fragile structure and weak mechanical properties of this type of system. We successfully prepared cryogels made of newly synthesized kefiran exopolysaccharide-chondroitin sulfate (CS) conjugate via carbodiimide-mediated coupling to overcome these drawbacks. The freeze-thawing procedure of cryogel preparation followed by lyophilization is a promising route to fabricate polymer-based scaffolds with countless and valuable biomedical applications. The novel graft macromolecular compound (kefiran-CS conjugate) was characterized through ^1^H-NMR and FTIR spectroscopy—which confirmed the structure of the conjugate, differential scanning calorimetry (DSC) and thermogravimetric analysis (TGA)—which mirrored good thermal stability (degradation temperature of about 215 °C) and, finally, gel permeation chromatography–size exclusion chromatography (GPC-SEC)—which proved an increased molecular weight due to chemical coupling of kefiran with CS. At the same time, the corresponding cryogels physically crosslinked after the freeze-thawing procedure were investigated by scanning electron microscopy (SEM), Micro-CT, and dynamic rheology. The results revealed a prevalent contribution of elastic/storage component to the viscoelastic behavior of cryogels in swollen state, a micromorphology with micrometer-sized open pores fully interconnected, and high porosity (ca. 90%) observed for freeze-dried cryogels. Furthermore, the metabolic activity and proliferation of human adipose stem cells (hASCs), when cultured onto the developed kefiran-CS cryogel, was maintained at a satisfactory level over 72 h. Based on the results obtained, it can be inferred that the newly freeze-dried kefiran-CS cryogels possess a host of unique properties that render them highly suitable for use in tissue engineering, regenerative medicine, drug delivery, and other biomedical applications where robust mechanical properties and biocompatibility are crucial.

## 1. Introduction

Tissue engineering and regenerative medicine (TERM) is a multidisciplinary science that focuses on repairing and replacing damaged tissues, or whole organs, in the body. This field aims to improve the treatment of various medical conditions, including injuries, genetic disorders, and degenerative diseases. This can involve using or combining cells, scaffolds, and growth factors [1]. Hydrogels in TERM have gained significant attention due to their ability to mimic the complexity of tissue defects and support cell colonization. They are composed of a network of polymer chains suspended in water and are similar to gel in their structure and consistency, but they can hold significant amounts of water [2]. Hydrogels are helpful for various applications, such as drug delivery, medical dressing, and tissue engineering [3]. One of the main advantages of hydrogels is their capability to recreate the complexity of a tissue defect in situ. In fact, synthetic polymers have been widely used in biomedical applications but often lack natural biopolymers’ inherent biocompatibility and bioactivity.

Moreover, exploring natural biopolymers/materials presents economic and environmental advantages [4]. Three-dimensional hydrogels based on natural polymers have become popular in the TERM field due to their unique properties, making them well-suited for use as a microenvironment for cell colonization [5]. However, specific crucial criteria will define the adequate characteristics of the device to fulfill the requirements of a biomaterial for medical uses, such as biocompatibility, elasticity, and mechanical strength. Furthermore, natural hydrogels derived from protein and polysaccharides have excellent biocompatibility, biodegradability, remodeling, and mechanical properties, making these biomaterials a promising strategy in TERM [6].

Chondroitin sulfate (CS) is a sulfated polysaccharide commonly linked to proteins as part of a proteoglycan. It is often used in TERM, as it is a key component of cartilage and other connective tissues. In addition, it has been used as a biodegradable polymeric vehicle for controlled drug delivery. This polymer, alone or in combination, is widely used as a therapeutic intervention for joint disorders treatment in the arthritis context since it represents an essential component of the proteoglycan aggrecan, an essential component of cartilage tissue. In addition, this polysaccharide has several advantageous properties, such as being highly versatile, readily available, biocompatible, and biodegradable [7]. Recently, it has been shown that the use of bacterial polysaccharides has demonstrated an increased interest in designing new biomaterials [8]. Kefiran, a polysaccharide from mainly lactic acid bacteria, is found in the flora of kefir grains. These grains are white or yellowish, translucent substances which form a gel-like matrix in water. Kefiran exopolysaccharide has several interesting properties, including good biocompatibility and biodegradability, making it a potential candidate for biomedical applications. Recently, Kefiran-based hydrogels were produced using a freeze gelation technique [9]. Though, some of the biopolymer-based hydrogels demonstrated a particular weakness and, more importantly, lacked adequate mechanical properties that can be improved by different crosslinking reactions [10]. Nevertheless, the application of these natural-based hydrogels is principally constrained by their fragile structure and poor mechanical properties, among other limitations. Thus, chemical coupling methods are currently being studied to overcome these shortcomings and enhance the polymer’s properties.

Cryogelation is a relatively straightforward and emerging technique used to produce porous and lightweight scaffolds with interconnected pore networks, which are beneficial for many applications, including tissue engineering and drug delivery. This procedure provides the ability to create scaffolds with controlled pore size and shape, which can be tailored to the particular needs of several tissue types. In addition, the cryogelation technique can create multi-layered scaffolds, which can mimic the complexity of natural tissue structures and promote tissue integration [11]. Altogether, due to their unique properties, cryogels have been used for cell delivery, drug delivery, cancer immunotherapy, tissue engineering, bioseparations, biosensors, and cell culture in three dimensions. Utilizing cryogelation as a technique for fabricating scaffolds in tissue engineering and regenerative medicine (TERM) applications offers several advantages, including simplicity, cost-effectiveness, scalability, reproducibility, versatility, and the ability to precisely control the molecular weight, composition, and structure of the resulting materials. However, this technique has certain limitations to consider when designing and producing scaffolds. One of the key limitations is that cryogelation can only be used with certain types of polymers, such as gelatin, alginate, and chitosan, which can form a gel network upon cooling. This limits the range of materials that can be used to produce scaffolds using cryogelation. Another limitation is the potential for ice crystal formation during freezing, which can cause damage to the scaffold structure and compromise its mechanical strength. Finally, the process of cryogelation can be time-consuming and requires careful control of various parameters such as freezing rate, thawing conditions, and crosslinking density to achieve the desired properties of the scaffold. Despite these limitations, cryogelation remains a valuable technique for producing scaffolds for biomedical applications, and ongoing research is focused on improving the process and addressing its limitations [12]. In recent years, numerous studies have highlighted the effectiveness of cryogelation techniques for producing various types of natural polymer-based materials. For instance, some studies have demonstrated the successful use of cryogelation for producing chondroitin sulfate-based cryogels [13], while others have focused on developing biodegradable films from kefiran-based cryogel systems [14]. Building upon these promising findings, our research aims to leverage the unique properties of these two natural polymers to create a novel cryogel system that can be applied in various biomedical applications. By combining the advantages of chondroitin sulfate-based and kefiran-based cryogels, we aim to develop a highly porous, mechanically stable, and biocompatible material that can be used for tissue engineering, drug delivery, and other biomedical applications.

In the current study, we successfully synthesized a novel biomaterial by chemically modifying the kefiran exopolysaccharide with chondroitin sulfate through a carbodiimide-mediated coupling reaction. In addition, we fully characterized the developed conjugated kefiran-CS material using ^1^H-NMR and FTIR spectroscopy, DSC, and GPC-SEC. The obtained 3D cryogels based on kefiran-CS were investigated through SEM, Micro-CT, and rheology. The cytotoxicity screening was evaluated by seeding human adipose-derived stem cells onto the conjugated kefiran-CS cryogels.

## 2. Materials and Methods

### 2.1. Materials

Kefiran is a water-soluble polysaccharide composed of equal amounts of galactose and glucose. As previously described, kefiran exopolysaccharides were extracted from kefir grains [15]. Briefly, the grains were heated in 200 mL of H_2_O at 80 °C for 30 min. After centrifugation at 18,300× *g* for 20 min at 20 °C, the exopolysaccharide in the supernatant was precipitated overnight in two volumes of absolute cold ethanol at −20 °C. After new centrifugation at 18,300× *g* for 20 min at 4 °C in H_2_O, the pellets were dissolved at 60 °C, and an additional round of ethanol precipitation was achieved. The pellets were then dissolved in H_2_O at 60 °C and concentrated for a crude polysaccharide by freeze-drying.

Regarding the chondroitin sulfate (CS), this polysaccharide is a linear polysaccharide with a structure based on (1–3)-β-N-acetyl-d-galactosamine and (1–4)-β-glucuronic acid; the chondroitin sulfate A sodium salt from the bovine trachea (cat 39455-18-0, 27042), used for this research, was provided by Sigma-Aldrich (St. Louis, MO, USA).

### 2.2. Synthesis and Characterization of Conjugated Kefiran-Chondroitin Sulfate

The conjugated kefiran-chondroitin sulfate solution was prepared based on a slightly modified procedure of carbodiimide-mediated coupling [16]. Briefly, the chondroitin sulfate was conjugated with kefiran according to the following steps: First, a solution of MES buffer (pH 4.5, 0.1 mol/L) was prepared by dissolving 1.95 g of methanesulfonic acid ≥ 99.5% (MES) (Sigma-Aldrich, St. Louis, MO, USA) in 100 mL of distilled water. Kefiran (0.1 g) was then dissolved in 100 mL of the MES buffer (pH 4.5). Then, N-(3-Dimethylaminopropyl)-N′-ethylcarbodiimide hydrochloride (EDC) (Sigma-Aldrich, St. Louis, MO, USA) (3.20 mg) and sulfo-NHS (N-Hydroxysulfosuccinimide) sodium salt (Sigma-Aldrich) (1.60 mg) were dissolved in 5 mL of MES buffer (pH 4.5). This solution was then mixed and stirred for 15 min, with the first solution of kefiran (solution A). Next, chondroitin sulfate (0.1 g) was dissolved in 10 mL of MES buffer (pH 4.5) and added to an EDC solution (1.3 mg in 5 mL of MES buffer) (solution B). Solution A (the kefiran mixture) and solution B (the CS mixture) were stirred for 24 h. The final solution was purified by dialysis for 1 week. The obtained solution, kefiran-CS conjugate, was lyophilized at −78.9 °C and 0.035 mbar for 7 days (Telstar LyoAlfa 10/15).

### 2.3. Preparation of Freeze-Dried Kefiran-Chondroitin Sulfate Cryogel

The conjugated kefiran-CS solution 2% *w*/*v* was prepared with ultrapure H_2_O. The solution was transferred to a 96-well microplate stored immediately at −2 °C for 24 h and then thawed at 4 °C for 24 h. The kefiran-CS cryogels in a dry state were obtained by freeze-drying the hydrogels (Telstar LyoAlfa 10/15) for 7 days (at −77.8 °C and 0.039 mbar).

### 2.4. Methods

#### 2.4.1. Physicochemical Characterization of Kefiran-Chondroitin Sulfate Material

- ^1^H-NMR

The kefiran-CS conjugate was solubilized in deuterated water (1 mg/mL) at room temperature, and 700 µL of this solution was transferred into an NMR tube [15]. The NMR spectra were obtained on a Bruker AVANCE 400 spectrometer at 60 °C, using a resonance frequency of 400 MHz. The software MestReNova 9.0 (Mestre-lab Research) was used to process and analyze the obtained spectra to assess the modification, as well as the estimation of the degree of substitution (DS), which corresponds to the fraction of modified hydroxyl groups per repeating unit.

- FTIR

The transmission spectra were acquired on a Shimadzu-IR Prestige-21 spectrometer (Shimadzu, Kyoto, Japan), equipped with attenuated total reflectance (ATR) crystal, using 32 scans, a resolution of 4 cm^−1^, and a wavenumber range between 4000 and 600 cm^−1^ [17]. The freeze-dried samples were pressed through ATR crystal to analyze in transmission mode and acquire the data.

- GPC-SEC

The molecular weight (Mw) of the kefiran-CS conjugate was determined by performing GPC-SEC of 1 mg/mL solutions prepared in phosphate-buffered saline (0.01 M phosphate buffer, 0.0027 M potassium chloride and 0.137 M sodium chloride, pH 7.4, at 25 °C) and 0.05% *w*/*v* NaN_3_ at a flow rate of 1 mL/min. GPC measurements were performed on a Malvern Viscotek TDA 305 (Malvern Panalytical, Malvern, UK) with a refractometer Bischoff RI-Detector 8110 (Elsichrom, Knivsta, Sweden), right- and low-angle light scattering (LS), and viscometer detectors on a set of four columns: pre-column Suprema, 5 µm, 8 × 50; Suprema 30 Å, 5 µm, 8 × 300; and Suprema 1000 Å, 5 µm, 8 × 300. The system was kept at 30 °C. The absolute molecular weight was determined after calibration of the Refractive Index (RI) and Light Scattering (LS) detectors performed by using a pullulan with a number-average molecular weight (Mn) of 48.8 kDa and a polydispersity index (PDI) of 1.07. In addition, the refractive index increment (dn/dc) specific to the polysaccharide was set to 0.15 [17].

- DSC

DSC experiments were conducted using TA-Q100 equipment (TA Instruments, New Castle, DE, USA) under a nitrogen atmosphere. Kefiran-CS conjugate (5–10 mg of lyophilized cryogel) was filled into aluminum pans [15]. An empty aluminum pan was utilized and ran simultaneously as DSC reference. In a nitrogen atmosphere, the samples were heated in two phases from 0 °C to 250 °C (heating rate of 20 °C/min) and cooled back to 0 °C by performing two such heating-cooling cycles with holding the samples at 250 °C for 2 min.

- STA

The STA investigation was carried out on the Simultaneous Thermogravimetric Analyzer (STA7200 Series, Hitachi High-Tech Corporation, Hitachinaka, Japan), providing TGA thermograms with a heating rate of 10 °C/min in the range of 30–600 °C [15]. To do that, samples of conjugated kefiran-CS powder (3.36 mg) were put in an alumina pan in an atmosphere of dry nitrogen.

#### 2.4.2. Characterization of Freeze-Dried Kefiran-CS Cryogel

- Micro-computed tomography (micro-CT) analysis

The microstructure of the kefiran-CS cryogel after freeze-drying was assessed using a high-resolution X-ray microtomography equipment Skyscan 1272 scanner (Bruker Micro-CT, Kontich, Belgium) with a defined pixel size of 5 μm, a 2452 × 1640 resolution, and a rotation step of 0.45° over a rotation range of 360° [17]. X-ray exploration of the dry gel’s microstructure was performed with the X-ray source voltage of 50 keV of energy and a current intensity of 200 μA with no filter. After data acquisition, the reconstructed grey-scale images were converted into binary images using a dynamic threshold of 33–255. The binary images were used for morphometric analysis (CT Analyzer v1.12.0.0, SkyScan, Kontich, Belgium) by quantifying porosity, mean pore size, and mean wall thickness. The image processing and reconstruction software Data Viewer (v1.6.6.0) and CT-Vox (v2.0.0) (SkyScan, Kontich, Belgium) were used to create, visualize, and register 3D virtual models and cross-sectional and longitudinal cuts.

- Scanning electron microscopy (SEM) analysis

The kefiran-CS cryogel, after freeze-drying, was fixed to aluminum stubs (with carbon tape) and platinum-coated in a sputter coater (EM ACE600, Leica, Wentzler, Germany) [9]. The morphology images were obtained on a JSM-6010LV SEM (JEOL USA Inc., Peabody, MA, USA), featuring integrated energy dispersive spectroscopy (EDS) (INCAx-Act, PentaFET Precision, Oxford Instruments, Abingdon, UK).

- Rheology of Kefiran-CS cryogels

A Kinexus pro + rheometer (Malvern Panalytical, Malvern, UK) equipped with a 316-grade stainless steel cone-plate system, comprised an upper measurement geometry cone (4° angle and 40 mm of diameter) and a lower pedestal plate, was used for rheological analyses using the acquisition software rSpace (version 1.76) [9].

Oscillatory experiments were performed on the conjugated kefiran-CS cryogels, to study their viscoelasticity frequency sweep curves were obtained at 37 °C from 0.01 Hz to 1 Hz with a shear strain of 1.3%. The conjugated kefiran-CS cryogel, previously freeze-dried, was moistened prior to the oscillatory experiments. All plots were obtained by the average of at least 3 experiments. Through oscillatory experiments, the average mesh size (*ξ*, nm) and the crosslinking density (n*_e_*, mol/m^3^) of the scaffolds were determined [18]. The mesh size is defined as the distance between the crosslinking points that can be established by the rubber elastic theory (RET), Equation (1):

(1) $$\xi ={\left(\frac{G^{\prime }N_{A}}{RT}\right)}^{-1/3}$$

where *G*′ is the storage modulus, *N_A_* is the Avogadro’s constant (6.022 × 10^23^), *R* represents the molar gas constant (8.314 J/K mol), and *T* is the absolute temperature (25 °C = 298.15 K). The n_e_ quantity (mol/m^3^) is defined by the number of elastically active connection points in the network per unit of volume, calculated by RET, Equation (2):

(2) $$n_{e}=\frac{G_{e}}{RT}$$

where *G_e_* is the plateau value of storage modulus measured by the frequency sweep test [19].

#### 2.4.3. Cytotoxicity Evaluation of Freeze-Dried Kefiran-CS Cryogel

- Human adipose-derived stem cells (hASCs) isolation and culture

The cells were obtained from human adipose tissue after the liposuction procedure, performed at Hospital da Prelada (Porto), after the patient’s informed consent, and under a collaboration protocol approved by the ethical committees of both institutions. The hASCs were isolated, employing a previously established methodology [20].

The cytotoxicity of kefiran-CS cryogel, after freeze-drying, was analyzed using human adipose-derived stem cells (hASCs) [9]. To achieve that, the cells were seeded on top of each scaffold at a density of 1 × 105 cells/cm_2_ and supplemented with 10% fetal bovine serum (FBS; Life Technologies, Carlsbad, CA, USA) and 1% antibiotic–antimycotic (Life Technologies, Carlsbad, CA, USA). Cultures were kept under a humidified atmosphere of 5% (*v*/*v*) CO_2_ at 37 °C.

- Cell viability assay

The hASCs’ viability was assessed using the AlamarBlue^®^ assay (Bio-Rad, Amadora, Portugal). AlamarBlue^®^ has resazurin, which is reduced to resorufin upon entering metabolically functional cells, resulting in a highly fluorescent compound that can be easily detected as an indicator of living cells. At 24 h, 48 h, and 72 h, the freeze-dried kefiran-CS cryogel developed was incubated with 10% (*v*/*v*) of AlamarBlue^®^ reagent in DMEM for 4 h at 37 °C. The supernatant was then transferred to a 96-well black plate, and fluorescence measurements were carried out using a Biotek Synergy HT Microplate Reader (Marshall Scientific, Hampton, VA, USA) with Ex/Em at 530/590 nm. Then, total double-stranded DNA (dsDNA) was quantified to evaluate the cells’ proliferation. First, the scaffold was recovered at each time point, incubated for 1 h at 37 °C in ultrapure H_2_O, and stored at −80 °C until analyzed. The scaffold in ultrapure H_2_O was then sonicated for 15 min and used for dsDNA quantification, utilizing the kit of Quant-iT PicoGreen dsDNA (Invitrogen, Carlsbad, CA, USA) according to the manufacturer’s instructions. Briefly, samples were transferred to a 96-well white plate and diluted in TE buffer. After adding the Quant-iT PicoGreen dsDNA reagent, samples were incubated (RT, 10 min) in the dark and fluorescence was quantified using a microplate reader with Ex/Em at 480/530 nm. The values of the relative fluorescence unit were converted into ng/mL using a standard DNA curve in the 1–2000 ng/mL of dsDNA range.

#### 2.4.4. Statistical Analysis

For data analysis, a two-way ANOVA followed by Tukey’s multiple comparisons test was achieved using GraphPad Prism version 8.0.1 for Windows (GraphPad Software, San Diego, CA, USA) to identify statistically significant differences between sample groups. The data are presented as mean ± standard deviation.

## 3. Results and Discussion

### 3.1. ^1^H Nuclear Magnetic Resonance Spectroscopy (NMR)

**Figure 1:**
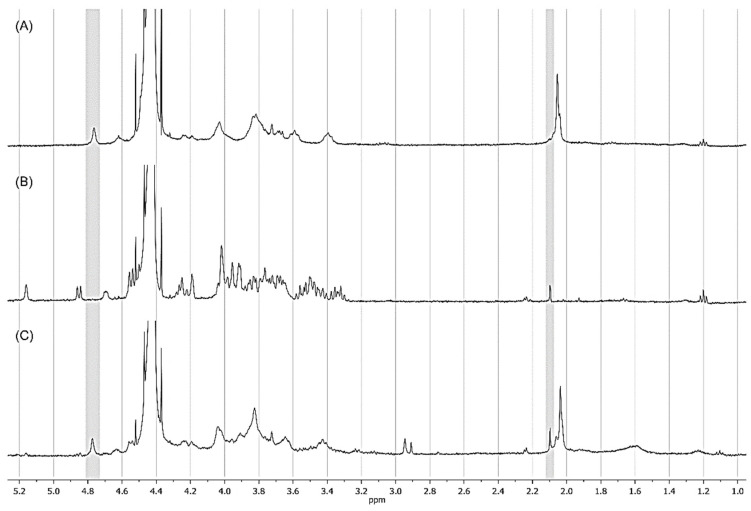
^1^H-NMR spectra of (**A**) chondroitin sulfate, (**B**) Kefiran, and (**C**) Kefiran-CS conjugate in D_2_O at 60 °C.

Proton NMR spectroscopy is a powerful tool for characterizing complex carbohydrates’ structure, composition, and function [21]. The obtained ^1^H-NMR spectra (in D_2_O at 60 °C) of Kefiran, chondroitin sulfate (CS), and the synthesized kefiran-CS conjugate are shown in Figure 1.

The spectrum obtained for chondroitin sulfate (Figure 1A), which possessed a well-established and well-defined chemical structure, agreed with the findings reported in the previous literature, displaying a similar profile and confirming the accuracy of our research [22]. Moreover, the kefiran polysaccharide spectrum was already previously discussed (Figure 1B), showing a peak at 5.15 ppm for an anomeric α hydrogen, and three signals at the chemical shifts of 4.85 ppm (doublet), 4.67 ppm, and 4.62 ppm for several anomeric β hydrogens, assigned to sugar residues on lateral branches [15]. This further confirms the structural complexity of kefiran and its distinct chemical profile. Regarding the ^1^H-NMR spectrum of the developed kefiran-CS conjugate (Figure 1C), it confirmed the functionalization of kefiran by adding a chondroitin sulfate molecule to its structure. In fact, the appearance of a distinctive peak that corresponds to δ 4.77 ppm corresponds to the ^1^H signal of the 4-sulfated site of the galactosamine unit of chondroitin sulfate on the kefiran-CS conjugate spectra.

The degree of substitution (DS) is well-defined as a measure of the degree of modification in a molecule. It is the ratio of the number of substituent groups per monomer unit commonly used to quantify the extent of chemical modification, such as the degree of sulphation in the newly developed kefiran-CS conjugate. The DS will depend on the structure and reaction in question [23]. In our research, the kefiran-CS conjugate had a degree of substitution (DS) of 15.74%. In this pioneering study, a carbodiimide-based modification was performed involving kefiran and chondroitin sulfate. Previous studies on the chemical modification of kefiran were carried out by methacrylating kefiran with methacrylic anhydride, resulting in a higher degree of substitution (about 48%) [24] compared to the new kefiran-CS conjugate. More recently, kefiran was esterified with octenyl succinic anhydride, resulting in a shallow degree of substitution (about 0.041%) [25]. Further studies could be explored to increase the degree of substitution by changing the concentration of the modifier used in the reaction or prolonging the reaction time.

This value was calculated by taking the ratio of the average intensity of the signal of protons belonging to the 4-sulfated sites of the galactosamine units to the average intensity of the kefiran polysaccharide’s methyl protons at δ 2.1 ppm.

It is worth noting that chemical modification is a widely used method to improve a polymer-based material’s physicochemical and biological properties. Furthermore, grafting other groups onto the macromolecular backbone can significantly enhance its performance. Sulphation is the most usual modification of proteoglycan chains, which plays a critical role in regulating the physiological functions of proteoglycans in developing tissues. Moreover, it is well-known that sulfate groups play an important role in several biological processes, such as regulating inflammation, blood coagulation, and cell growth. They are also identified to enhance the bioactivity of polysaccharides, making them worthwhile in TERM applications [26].

### 3.2. Fourier Transform Infrared Spectroscopy (FTIR)

FTIR spectroscopy has proved to be a very useful tool to help characterize materials by investigating polymer components’ structure and dynamics during some chemically induced possible transformations [27].

**Figure 2:**
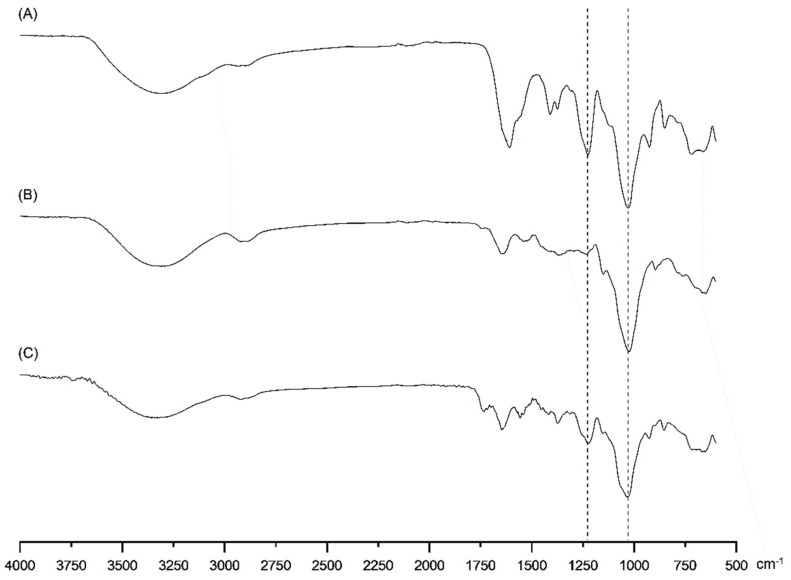
FTIR spectra of (**A**) CS and (**B**) Kefiran, (**C**) Kefiran-CS conjugate.

FTIR analysis successfully confirmed the obtaining of kefiran-CS conjugate (Figure 2).

Comparing infrared spectra of chondroitin sulfate, kefiran, and conjugated kefiran-CS material allowed us to find that they shared some typical peaks around 3400 cm^−1^, corresponding to stretching vibrations of O–H and N–H bonds [28]. The band identified at around 2900 cm^−1^ is assigned to CH_3_ and –CH_2_– groups [29], and those observed in the region 1700–1300 cm^−1^ could be attributed to the stretching vibrations of C=O bonds and a variety of bending modes associated with C–H (in –CH_3_ groups), O-H (in -OH groups), or N-H (in N-acetyl groups) bonds. This region offers relevant information on structural features of polysaccharides useful in their industrial applications, as reported elsewhere [30]. It is essential to highlight that the peak, inside the fingerprint region (600–1500 cm^−1^) around 1225 cm^−1^ corresponding to stretching vibrations of S=O bonds in sulfate groups, was observed for CS and kefiran-CS conjugate spectra but was not present in kefiran spectrum [31]. Furthermore, Figure 2 shows that all polymers shared a peak around 1030 cm^−1^, assigned to glycosidic (C-O-C) linkage vibrations superimposed on symmetric stretching vibrations of S=O bonds in sulfate groups [32]. In our research, by grafting kefiran to CS, the conjugate kefiran-CS must possess ester groups (freshly synthesized) by which the two initially separated molecules of kefiran and CS (prior to coupling reaction) will be chemically linked together into the final product. Indeed, a weak absorption peak located at about 1750 cm^−1^, assigned to the stretching vibration of C=O in ester groups, is visible on the FTIR spectrum of the kefiran-CS conjugate (Figure 2C), proving the synthesis of this macromolecular product. Thus, the successful grafting of chondroitin sulfate to kefiran developed in this research was confirmed by combining the information obtained by FTIR and ^1^H-NMR spectroscopy.

It is important to point out that the presence of sulfated groups in the backbone of a polysaccharide, such as kefiran, can provide several benefits for biomedical and biotechnological applications, due to their numerous therapeutic properties. These include enhanced bioactivity, improved binding to proteins and cells, and increased stability. Moreover, incorporating sulfated groups can result in a polysaccharide with specific bioactivities, which can be useful for applications. The increased binding to proteins and cells can also be helpful in these applications, as well as for wound healing. Overall, incorporating sulfated groups can enhance the properties and functions of a polysaccharide and improve its potential for a broad range of applications [33].

### 3.3. Gel Permeation Chromatography–Size Exclusion Chromatography (GPC-SEC)

In our study, GPC-SEC was used to determine the molecular weight distributions of the Kefiran, CS, and chemically modified kefiran polymer, with high accuracy. This technique is considered a critical method to analyze a polymer’s molecular weight that could be a candidate for TERM application. This information is an important parameter since the properties of a polymer, such as its solubility and mechanical strength, depend on its molecular weight [34]. Table 1 shows the values of the average molecular weights (weight average and number average molecular weight) and the corresponding values of the polydispersity index (PDI) for the investigated systems.

In this study, the results showed a longer polymer chain with the development of a higher molecular weight of the resulting kefiran-CS polymer, due to the carbodiimide-mediated coupling reaction between the two individual polysaccharides. Moreover, the PDI value of the final kefiran-CS conjugate is almost equal to one, indicating that the compound polymer chains are roughly uniform in size. In fact, these peculiarities (molecular weight and PDI) of conjugate kefiran-CS can significantly influence its performance, such as viscosity, solubility, and mechanical properties, which can in turn, determine its potential applications. Furthermore, changing the molecular mass of a polymer through physical/chemical approaches may improve its bioactivities, which may induce adequate properties required for biomedical applications [35]. In fact, high molecular weight polymers typically have better mechanical properties, while low molecular weight polymers may be more easily processed and have better solubility. Therefore, understanding the molecular weight of natural polymers is essential for tailoring their properties to specific biomedical applications [36].

Natural polymers are characterized by weak mechanical properties, such as fragility and low impact strength, which can limit their potential uses. Chemical modification of natural polymers, a vast domain in polymer science, represents an attractive alternative route to synthetic polymers for engendering biodegradable polymers. In fact, blending two or more biopolymers enhances the physical and mechanical properties of natural polysaccharides by reinforcing the polymer’s matrix with other biodegradable polymers [37]. The coupling of kefiran with chondroitin ssulfate resulted in the production of cryogels with increased viscosity and mechanical strength, as demonstrated by their ability to recover their shape under loading conditions. These improved properties are particularly relevant for TERM applications, where the gel-like 3D structure replaces or repairs tissues requiring high/adequate mechanical performances. Usually, a natural polysaccharide, such as chondroitin sulfate, will be grafted with vinyl monomers to develop biodegradable materials with improved properties for specific applications [38]. To the best of our knowledge, the modification of kefiran exopolysaccharide performed in our research by simple chemical grafting with chondroitin sulfate, has never been performed.

### 3.4. Differential Scanning Calorimetry (DSC)

**Figure 3:**
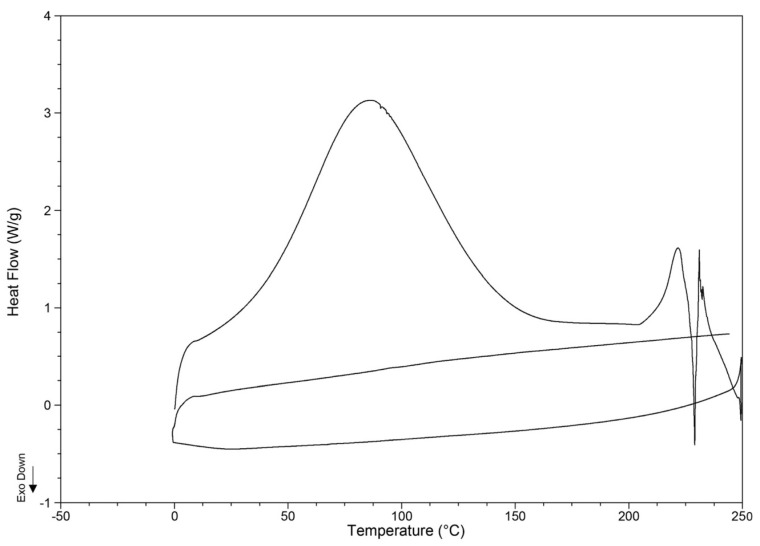
Differential scanning calorimetry thermal profile of Kefiran-CS conjugate in a dry state by DSC.

Thermal stability may be defined as the capability of a material to withstand molecular cracking under thermal stress. The well-defined endothermic peak at 87.6 °C for kefiran-CS conjugate suggests the existence of a phase transition during sample heating (Figure 3). This change is most likely a melting process of the microcrystalline regions formed when the initial solution of conjugate kefiran-CS was frozen at −20 °C for 24 h as the first part of the cryogel preparation according to the freeze-thaw procedure.

Practically, these microcrystalline regions are physical cross-linkages, resulting from favorable attractive intra and intermolecular interactions, mainly mediated by hydrogen bonding between the polymer chains of the kefiran-CS polymer. At temperatures above 215 °C, the thermogram shape reveals a complex process of decomposition of the kefiran grafted with CS (Figure 3).

The thermal behavior of polymers is directly related to the bond energies in the polymer structure, involving noncovalent or/and covalent interactions, among others. Moreover, the parameters associated with the polymer structure, such as the molecular weight, the nature of functional groups, and the degree of branching and crosslinking, substantially alter its thermal behavior [39,40].

It is essential to highlight that the grafted kefiran polymer with chondroitin sulfate showed better thermal stability than that of its native form, for which a much lower decomposition temperature (156 °C) was experimentally found [17]; thus, the onset temperature of the degradation of the kefiran-CS conjugate seems to be enhanced to a value of about 215 °C by functionalizing kefiran with chondroitin sulfate to eventually synthesize a new material with a higher thermal stability.

### 3.5. Simultaneous Thermal Analysis (STA)

**Figure 4:**
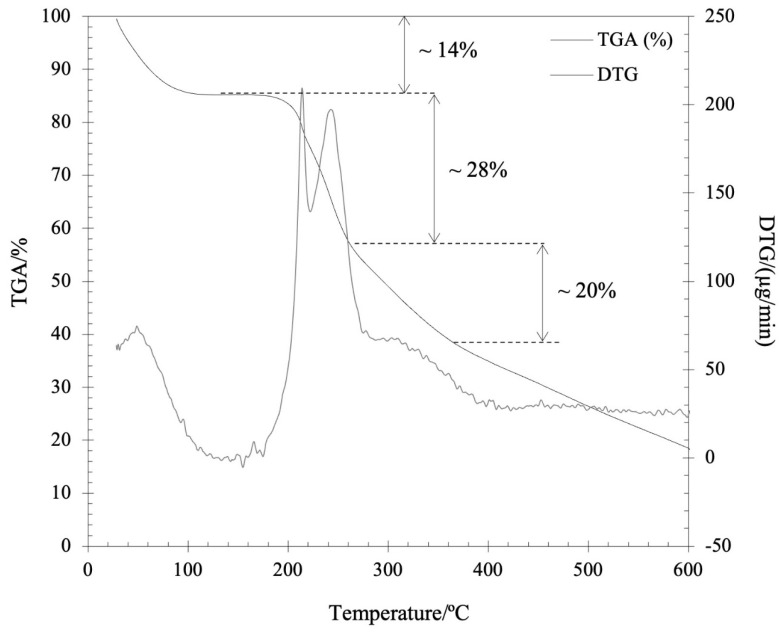
TGA and DTG curves of conjugate Kefiran-CS (30 °C to 600 °C).

The TGA of the kefiran-CS conjugate (sample weight ~3.362 mg) was performed at a heating rate of 10 °C/min from 30 to 600 °C, under nitrogen purging (50 mL/min) (Figure 4). The first step of weight loss on TGA trace (ca. 14%) during heating from 30 to 87 °C was ascribed to moisture loss.

Two other overall stages of weight loss were detected with increasing temperature: the second stage (200–280 °C), consisting of two steps of mass loss (based on the existence of two peaks on DTG curve), corresponds to a total weight loss of 28%; and the third stage (280–360 °C) of 20% weight loss developed as a lower rate process. This multistage decomposition of the main chain structure of the kefiran-CS conjugate substantially differs from the single-step decomposition of kefiran previously reported [17]. At temperatures above 360 °C, the weight loss (fourth stage) was associated with sample mineralization. By comparison, the thermal behavior (DSC, TGA) revealed in this study for the newly synthesized kefiran-CS conjugate when compared with the data reported elsewhere for kefiran [17,36], showed a relatively similar thermal stability, despite the differences found in decomposition stage(s) of the two distinct compounds.

### 3.6. Assessment of the Freeze-Dried Kefiran-CS Cryogels’ Morphological Properties

Morphological properties of scaffolds refer to their physical characteristics, such as shape, size, and surface structure. These properties can significantly impact the scaffold’s ability to support tissue growth and repair, and must be carefully designed and optimized to match the specific requirements of the intended application [41].

**Figure 5:**
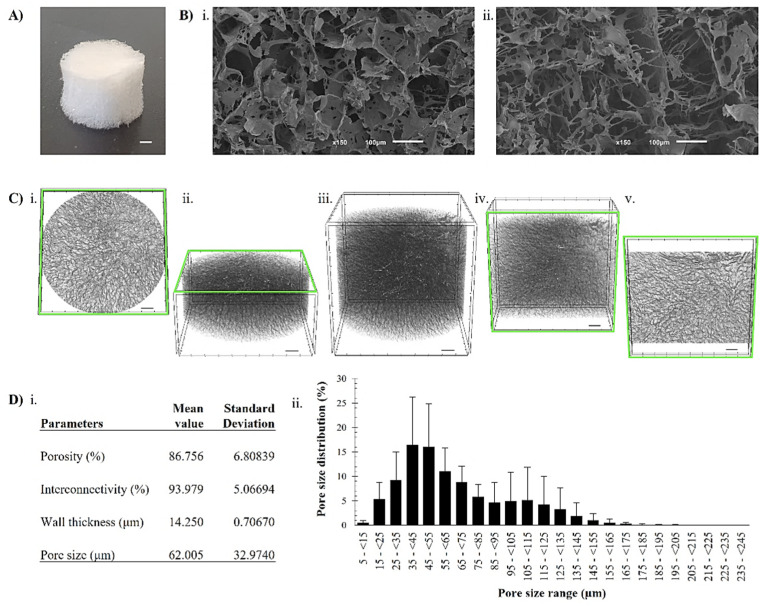
Morphology of kefiran-CS cryogels after freeze-drying. (**A**) Macroscopic image (scale bar: 1 mm); (**B**) SEM microstructure of surface at a magnification of ×150: longitudinal section (**i**); cross-section (**ii**); (**C**) 3D reconstructed kefiran-CS cryogels using micro-CT: cross-sectional (**i**,**ii**), longitudinal (**iv**,**v**) and fully reconstructed (**iii**) views (scale bars: 500 µm); and (**D**) micro-CT data: structure characterization (**i**) and pore size distribution (**ii**).

SEM imaging and Micro-CT (Figure 5), as imaging techniques, are commonly used to evaluate the scaffold’s microstructure by providing high-resolution two-dimensional images of the surface of specimens down to the nanometer scale [41]. Thus, the SEM investigation of freeze-dried kefiran-CS cryogels showed lightweight, porous materials with a sponge-like structure that were created during, and subsequent to, freeze-drying (Figure 5B). In fact, the freeze-dried cryogel exhibited an aerogel-like structure with a high surface area and low density, which can be advantageous for specific applications, such as tissue engineering and drug delivery [42]. Furthermore, this newly developed structure offers unique properties such as high porosity, large surface area, low density, and high mechanical strength, making it a promising material for various applications.

The morphometric analysis of the freeze-dried kefiran-CS cryogels (referred to in this section as cryogels) demonstrated their high porosity (87 ± 6.8%) and a widespread pore size distribution with a mean value of 62 μm (Figure 5D). Moreover, the cryogel’s porosity, calculated as the ratio of void volume to total volume, was similar to the modified methacrylated-kefiran scaffold previously produced by our research group [24]. Therefore, such a 3D scaffold must possess a highly porous structure with an open, fully interconnected geometry for providing a large surface area that will allow cell ingrowth and uniform cell distribution [43].

In comparison to the native kefiran cryogel (porosity of 82.335%, pore size of 49.1 μm, and wall thickness of 13.4 μm) [9], the newly developed freeze-dried kefiran-CS cryogel structure showed larger sized pores. It is essential to highlight that a scaffold with larger pores is better for cell migration and penetration, thus is foremost for better tissue growth and formation [43]. In fact, designing the hydrogel network is critical since a scaffold with a high porosity easily allows the diffusion of oxygen, nutrients, and waste removal from cells. Furthermore, in a tissue engineering context, the high porosity of kefiran-CS cryogel, as obtained in our research, can be a beneficial feature in tissue engineering; it can allow for cell interconnectivity and infiltration [44]. The major purpose of producing an ideal 3D structure is to create an environment mimicking the native morphology of the extracellular matrix of the targeted tissue [45].

It is worth mentioning that the cryogelation technique (freeze-thaw followed by freeze-drying) used to develop freeze-dried kefiran-CS cryogel produces highly porous scaffolds. In this procedure, the water crystallization acts as a trigger enabling the subsequent gelation phenomena. Scaffold properties, such as its pore size, porosity, mechanical strength, degradation rate, and biocompatibility, depend mainly on the nature of the biomaterial and the production process, among others [46]. Several emerging techniques are being developed and tested to produce scaffolds used in TERM applications. These techniques include bioprinting, decellularization, electrospinning, and freeze-drying. They offer new possibilities for scaffold production in TERM and have the potential to improve the performance and functionality of scaffolds for various applications [47]. However, investigating alternative production techniques such as cryogelation is essential for broadening the scope of scaffold production for various purposes. This opens more options in selecting the methods that are best suited to individual requirements, resulting in even more advanced and efficient outcomes.

### 3.7. Assessment of the Kefiran-CS Cryogels’ Mechanical Properties

Our research evaluated the mechanical properties of the developed kefiran-CS cryogels. These 3D structures were wet before the rheological assays. In fact, the material must be suitable for the properties of the intended tissue or application. For example, more robust hydrogels are needed for weight-bearing tissues such as bones, while softer and more elastic materials are required for tissues such as skin [48].

**Figure 6:**
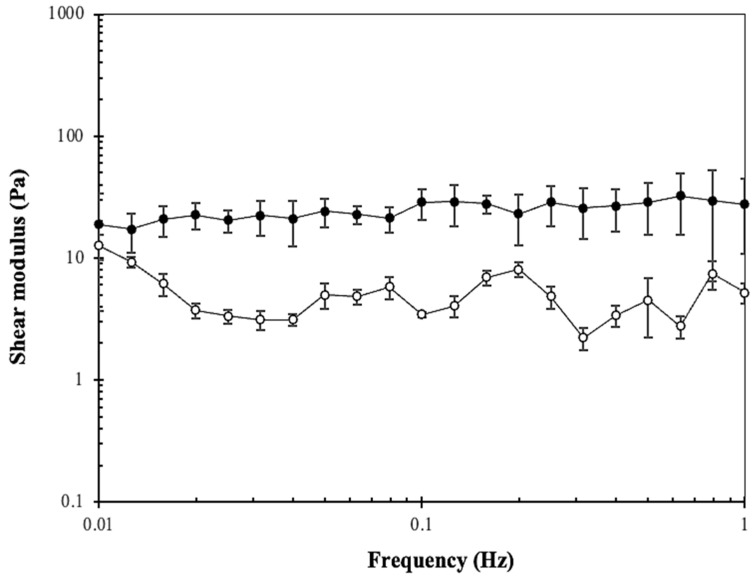
Mechanical analysis of Kefiran-CS cryogel (2% *w*/*v*) expressed as shear moduli vs. frequency (loss modulus G″—open symbols, storage modulus G′—filled symbol, shear strain—1.3%, temperature–37 °C).

The results presented in Figure 6 suggest that the kefiran-CS cryogel exhibited a prevalent elastic behavior, as the storage modulus is higher than the loss modulus. This means the material can return to its original shape after being subjected to external forces.

The viscoelastic properties of a material, such as its shock-absorbing capability, can be evaluated using phase angle (δ, 0° < δ < 90°) measurements [49]. This data relates to how much energy the material can dissipate during the impact. In fact, the phase angle of a hydrogel can provide important information about its mechanical behavior and response to applied forces. For instance, hydrogels with a large phase angle (i.e., highly viscoelastic) may be more deformation-resistant and have higher energy dissipation. In contrast, hydrogels with a small phase angle (i.e., more elastic) may be more prone to deformation and have a lower degree of energy dissipation [50].

Elasticity is the capacity of a material to deform under an applied force and regain its original shape when the force is removed. In the current research, the phase angle was about 11.17 ± 3.23°, showing an elastic behavior of the developed kefiran-CS cryogels. However, the phase angle of a previously modified methacrylated-kefiran scaffold was almost half what we observed in the current scaffold. This aspect reveals that the newly obtained kefiran-CS cryogels were more viscous than those of kefiran. Thus, the scaffold will take longer to respond to the applied strain, which could be interesting since a scaffold with a slow response time may offer benefits such as increased stability, durability, reduced damage, safety, and efficiency.

The carbodiimide-mediated reaction is a coupling method that uses carbodiimides to activate carboxylic acid groups to react with hydroxyl/amine groups belonging to the same or another type of polymer compound. This reaction can be employed to improve the mechanical and biological properties of natural polymers in the field of tissue engineering. Additionally, this coupling method can incorporate bioactive molecules such as growth factors or antibiotics into the polymer’s matrix, enhancing its therapeutic potential [51]. Furthermore, obtaining data on the kefiran-CS cryogel’s average mesh size (ξ/nm), crosslinking density (n_e_/(mol/m^3^)), and equilibrium storage modulus G_e_ is crucial for evaluating its mechanical strength and creating a hydrogel with the desired properties for tissue regeneration (Table 2).

The results show that the kefiran-CS cryogel has a higher mesh size, but has a lower storage modulus, and crosslinking density compared to the native kefiran scaffold. Notably, a smaller mesh size of a scaffold can result in a stiffer scaffold with better resistance to deformation, while larger mesh sizes can result in a softer scaffold with greater deformability. The adequate rheological characteristics have to be adapted to the specific requirements of the tissue being regenerated, such as the mechanical properties, cell attachment, and, tissue regeneration. The mechanical shear strength properties are important factors considered in designing and evaluating the scaffolds intended for use in TERM, as they can affect the stability and performance of the scaffold in vivo, and the response of the adherent cells to it [52].

### 3.8. Cytotoxicity Screening

**Figure 7:**
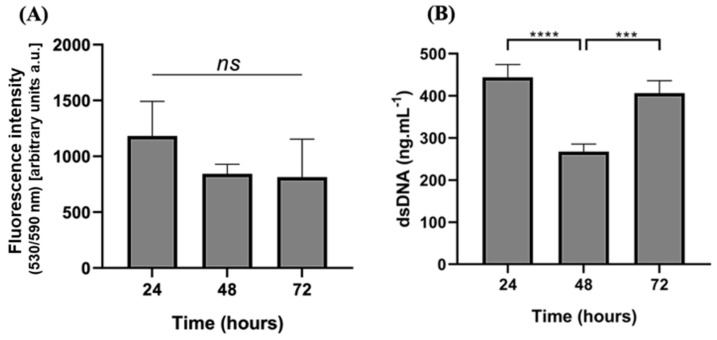
In vitro biological performance of Kefiran-CS cryogel. (**A**) hASCs’ metabolic function, and (**B**) DNA quantification upon cultured on freeze-dried Kefiran-CS scaffolds for 72 h. *ns* = not statistically significant. Symbols denote statistically significant differences at any timepoint of *p* < 0.05, where *** and **** corresponds to *p* < 0.0002 and *p* < 0.0001, respectively (n = 3).

The possible cytotoxicity was evaluated by performing the AlamarBlue^®^ assay (Bio-Rad, Amadora, Portugal). Results demonstrated that the metabolic activity of hASCs, when exposed to the developed freeze-dried Kefiran-CS porous scaffold (Figure 7), was maintained over 72 h of culturing.

dsDNA quantification was assessed to evaluate the proliferation of exposed hASCs and showed a significant decrease after 48 h. Still, after 72 h, cells recovered, proliferating to numbers similar to 24 h of culture. This could be justified because the cells have become accustomed to the cryogel as an adaptive response.

Adipose-derived stem cells (ASCs), a mesenchymal stem cell niche with multipotential differentiation and self-renewal property, have recently become the most attractive source for tissue engineering and regenerative medicine. In fact, these cells serve as a cell-based therapy due to the high availability of sources, easy accessibility, and relatively low ethical issues [53]. It is crucial to emphasize that the functionalized scaffolds not only preserve the hASCs’ metabolic activity but also enable the cells to adhere, proliferate, and form a 3D cellular microenvironment rather than being confined to a flat cell culture plate. Future studies will investigate the cytotoxicity of cells that are seeded within these cryogels over an extended period of time.

Finding an ideal hydrogel that fits all the requirements for a suitable biomaterial in all tissue engineering technologies is challenging. Promising scaffolds must tailor biological functions and remarkable mechanical properties and, mimic the cellular microenvironment in a controlled setting for cell growth, adhesion, and differentiation [54]. In the current research, the newly developed freeze-dried kefiran-CS cryogel was revealed to support the capability of hASCs to grow and to be metabolically active during the time of incubation; thus, we demonstrated that after coupling kefiran and CS into a new larger macromolecular structure (kefiran-CS conjugate), its biocompatibility remained satisfactorily and this newly synthesized polymer did not have any detrimental effect neither on cellular metabolic activity, nor cell proliferation.

## 4. Conclusions

In the current research, kefiran exopolysaccharide linked to chondroitin sulfate through carbodiimide-mediated coupling reaction was successfully synthetized, and its three-dimensional scaffolds were developed. While the chemical modification by linking the exopolysaccharide with the chondroitin sulfate molecule helps to resolve some shortcomings associated with weak mechanical properties of gels based on natural polysaccharide, it has also an inherent impact on the properties of the newly developed material. The results showed that adding functional groups, by linking kefiran and chondroitin sulfate molecules, changed the material’s molecular weight and enhanced the material’s mechanical strength, thermal stability, and other physical properties. Moreover, the new 3D structures were found to have no cytotoxic effects on the metabolic activity and proliferation of human adipose-derived stem cells that were seeded within it. The objective of the research was to address the limitations of natural polysaccharide-based cryogels, and the experimental findings suggest that the functionalized chondroitin sulfate/kefiran material exhibits improved structural, physicochemical, and biological properties, rendering it a promising candidate for biomedical applications.

## Figures and Tables

**Table 1:** Molecular weight distribution of Kefiran, CS, and Kefiran-CS conjugate.

Samples	Mw (kDa)	Mn (kDa)	PDI
Kefiran	810.64 ± 2.38	748.84 ± 29.48	1.082
CS	45.713 ± 0.23	33.84 ± 0.12	1.352
Kefiran-CS	1649 ± 4.52	1449 ± 1.23	1.138

**Table 2:** Rheological parameters of Kefiran-CS cryogels.

Sample	G_e/_Pa	*ξ*/nm	*n*_e_/(mol/m^3^)
Kefiran [17]	(99.6 ± 13.9) × 10^−4^	34.7 ± 1.7	0.040 ± 0.006
Kefiran-CS	(24.6 ± 4.2) × 10^−4^	55.5 ± 3.2	0.010 ± 0.002

## Data Availability

All data generated/analyzed throughout this research are included in this article.
